# Comparative gain-of-function effects of the *KCNMA1*-N999S mutation on human BK channel properties

**DOI:** 10.1152/jn.00626.2019

**Published:** 2019-12-18

**Authors:** Hans J. Moldenhauer, Katia K. Matychak, Andrea L. Meredith

**Affiliations:** ^1^Department of Physiology, University of Maryland School of Medicine, Baltimore, Maryland; ^2^Program in Neuroscience, University of Maryland School of Medicine, Baltimore, Maryland

**Keywords:** BK channel, calcium-activated potassium channel, *channelopathy*, human mutations, seizure

## Abstract

*KCNMA1*, encoding the voltage- and calcium-activated potassium channel, has a pivotal role in brain physiology. Mutations in *KCNMA1* are associated with epilepsy and/or dyskinesia (PNKD3). Two *KCNMA1* mutations correlated with these phenotypes, D434G and N999S, were previously identified as producing gain-of-function (GOF) effects on BK channel activity. Three new patients have been reported harboring N999S, one carrying a second mutation, R1128W, but the effects of these mutations have not yet been reported under physiological K^+^ conditions or compared to D434G. In this study, we characterize N999S, the novel N999S/R1128W double mutation, and D434G in a brain BK channel splice variant, comparing the effects on BK current properties under a physiological K^+^ gradient with action potential voltage commands. N999S, N999S/R1128W, and D434G cDNAs were expressed in HEK293T cells and characterized by patch-clamp electrophysiology. N999S BK currents were shifted to negative potentials, with faster activation and slower deactivation compared with wild type (WT) and D434G. The double mutation N999S/R1128W did not show any additional changes in current properties compared with N999S alone. The antiepileptic drug acetazolamide was assessed for its ability to directly modulate WT and N999S channels. Neither the WT nor N999S channels were sensitive to the antiepileptic drug acetazolamide, but both were sensitive to the inhibitor paxilline. We conclude that N999S is a strong GOF mutation that surpasses the D434G phenotype, without mitigation by R1128W. Acetazolamide has no direct modulatory action on either WT or N999S channels, indicating that its use may not be contraindicated in patients harboring GOF *KCNMA1* mutations.

**NEW & NOTEWORTHY** *KCNMA1*-linked channelopathy is a new neurological disorder characterized by mutations in the BK voltage- and calcium-activated potassium channel. The epilepsy- and dyskinesia-associated gain-of-function mutations N999S and D434G comprise the largest number of patients in the cohort. This study provides the first direct comparison between D434G and N999S BK channel properties as well as a novel double mutation, N999S/R1128W, from another patient, defining the functional effects during an action potential stimulus.

## INTRODUCTION

Channelopathy disorders are caused by the abnormal functioning of ion channel subunits (Kim 2014; Meredith 2015). The leading sources of channel dysfunction are de novo and inherited nucleotide changes, which can be classified as gain- or loss-of-function (GOF, LOF) mutations. GOF mutations alter channel activity in a way that increases current magnitude or duration, whereas LOF produces the opposite effect, to reduce current size or duration. Both types of mutations have been found in the *KCNMA1* gene (Bailey et al. 2019), which encodes the pore-forming α-subunit of the “Big K^+^” (BK) calcium- and voltage-activated channel (Dworetzky et al. 1994; McCobb et al. 1995; Pallanck and Ganetzky 1994). For the majority of *KCNMA1* mutations, it has not yet been established how the genetic changes alter BK channel function and under which conditions these alterations manifest (Bailey et al. 2019).

**Fig. 1.**
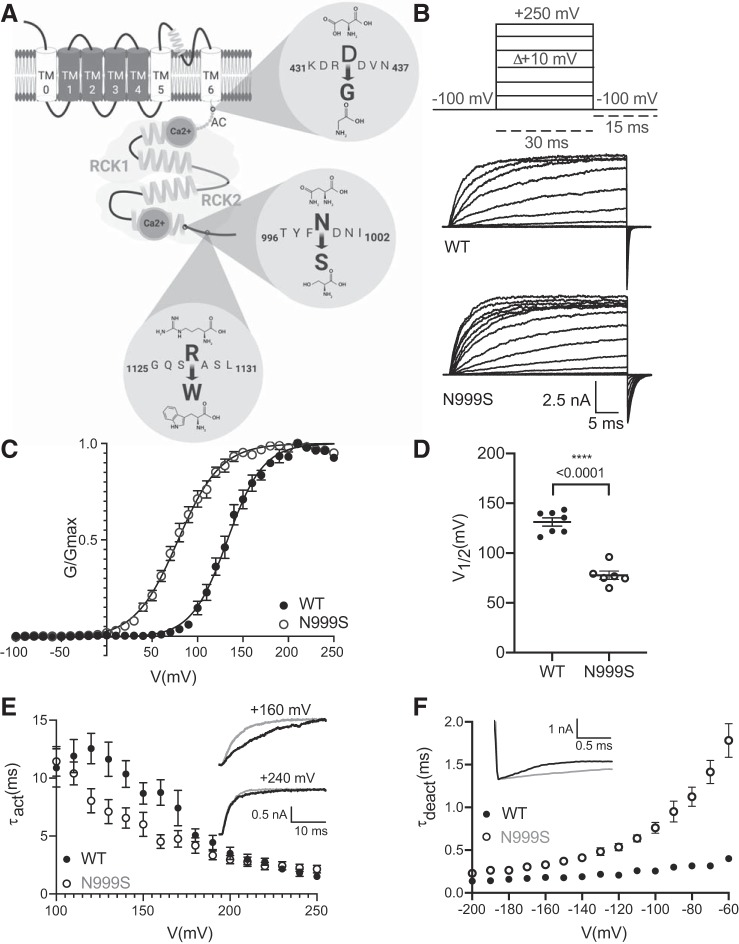
Wild-type (WT) and N999S BK channel properties in symmetrical K^+^ and 1 μM Ca^2+^. *A*: schematic representation of 1 BKα subunit, with the extracellular NH_2_ terminus, 7 transmembrane segments (TM0–TM6), and the intracellular COOH terminus containing the Ca^2+^ binding sites. Black, voltage sensor domain (VSD); gray, RCK1 and RCK2 domains comprising the gating ring. Dark gray circles correspond to the calcium binding sites, Gray dotted line between TM6 and RCK1 calcium binding site corresponds to AC region. Relative positions for the mutations D434G [13 known patients (Bailey et al. 2019; Du et al. 2005)], N999S [7 known patients (Bailey et al. 2019; Li et al. 2018; Zhang et al. 2015)], and R1128W [1 patient known (Heim et al. 2019)] are shown. The only patient carrying the mutation R1128W additionally harbors the N999S mutation. *B*: representative macroscopic currents at 20-mV increments with the voltage-step protocol applied. *C*: conductance-voltage (*G-V*) relationship for WT (*n* = 7) and N999S (*n* = 6) channels. *G* was normalized to the highest conductance calculated (*G*_max_). The *G-V* curves fit with a Boltzmann function (solid line). *D*: half-maximal voltage of activation (*V*_1/2_) values for WT and N999S channels (****P < 0.0001, 1-way ANOVA, Tukey post hoc test). *E*: activation time constant (τ_act_) vs. voltage for WT (*n* = 7) vs. N999S (*n* = 6). *Insets* show representative activation traces of both channels at +160 and +240 mV (the scale is the same for both). N999S shows significantly faster activation between +120 and +170 mV (*P* < 0.05, 2-way repeated-measures ANOVAs with Bonferroni post hoc) but not between −180 and +250. *F*: deactivation time constant (τ_deact_) for WT (*n* = 4) and N999S (*n* = 5) channels. N999S shows significantly slower deactivation kinetics compared with WT (*P* < 0.05 at −60 to −100, 2-way repeated-measures ANOVAs with Bonferroni post hoc). *Inset* illustrates representative portion of the tail currents at −60 mV (2 s of the total 20-s deactivation protocol).

BK channels have a characteristically large conductance (100–270 pS) (Latorre and Miller 1983) and a highly K^+^-selective pore. The channel is a homotetramer of four BKα subunits encoded by the *KCNMA1* gene (Meera et al. 1997; Shen et al. 1994). Each BKα subunit comprises seven transmembrane segments (TM0–TM6) and a large intracellular COOH terminus (Fig. 1*A*) (Meera et al. 1997). Within these regions, three domains are essential to activate and regulate channel activity. The voltage-sensing domain (VSD), located between TM1 and TM4, controls depolarization-dependent activation (Horrigan et al. 1999; Stefani et al. 1997). The COOH-terminal domain, called the “gating ring,” houses the regulators of K^+^ conductance domains (RCK1 and RCK2). Each RCK domain contains one high-affinity Ca^2+^-binding site, which enables channel opening in response to increases in intracellular Ca^2+^ (Hite et al. 2017; Sweet and Cox 2008; Xia et al. 2002). The third domain is the accessory subunit binding interface formed by the NH_2_ terminus and TM0, which is necessary for interaction with the accessory beta (β1–4) and/or gamma (γ1−4) subunits (Morrow et al. 2006; Wallner et al. 1996). Altogether, these three domains work together allosterically to open the pore gating domain (PGD) located between TM5 and TM6 and allow the K^+^ efflux from the cell (Horrigan and Aldrich 2002; Latorre et al. 2010).

Gating by voltage and Ca^2+^ confers specialized regulation of membrane potential in excitable cells. BK channels are expressed widely in neurons and muscle, where they exert specific effects on membrane potential through different splice variants, interactions with accessory subunits, and coupling to Ca^2+^ sources (Chen et al. 2005; Fagerberg et al. 2014; Gonzalez-Perez and Lingle 2019; Latorre et al. 2017). This selective tuning of BK channel properties through different molecular mechanisms and protein interactions produces distinct functional consequences for excitability. In the brain, the BK channel performs dual roles in regulating excitability depending on neuronal type (Contet et al. 2016; Montgomery and Meredith 2012). For example, BK channel activation can either deaccelerate (Purkinje neurons) or speed (GABAergic neurons) action potential (AP) firing, and therefore modulate neurotransmitter release (Contet et al. 2016; Latorre et al. 2017; Tseng-Crank et al. 1994). Thus BK channels manifest their pivotal role in preventing transmitter-related hyperexcitability, and therefore neuronal dysfunction, through this balance of activity (Bentzen et al. 2014).

Since the identification of the first disease-linked *KCNMA1* mutation (Du et al. 2005), the number of patients identified with neurological disorders and mutations in BK channels has increased (Bailey et al. 2019). Of the *KCNMA1* mutations currently known, only two are confirmed GOF with respect to their effects on BK channel activity, D434G and N999S (also identified as N995S and N1053S; Bailey et al. 2019; Du et al. 2005; Li et al. 2018; Zhang et al. 2015). These two mutations are located in the COOH-terminal gating ring (Fig. 1) and share a phenotypic association with epilepsy and/or paroxysmal nonkinesigenic dyskinesia (PNKD). The D434G mutation was identified in 13 related family members (Du et al. 2005), and the N999S mutation was found in 4 unrelated patients as a de novo mutation (Li et al. 2018; Wang et al. 2017; Zhang et al. 2015). Despite the GOF nature of both *KCNMA1* mutations, only the D434G mutation has been extensively biophysically characterized, whereas the N999S mutation has been studied in a more limited set of membrane conditions (Díez-Sampedro et al. 2006; Du et al. 2005; Li et al. 2018; Wang et al. 2017; Zhang et al. 2015).

In this study, we characterized the effects of the mutation N999S and the novel double mutation N999S/R1128W found in one patient (Heim et al. 2019) in the context of a brain-expressed *KCNMA1* splice variant (Shelley et al. 2013), under physiological K^+^ and Ca^2+^ conditions. Additionally, we compared the BK currents from channels containing the N999S or the D434G mutation to improve the understanding of the distinction between BK channel GOF mutations. Thus this study provides additional evidence for the changes in the BK channel’s biophysical properties induced by the N999S mutation, confirming its GOF nature in a different splice variant than previously characterized, in tandem with a second patient mutation, and in a new set of physiologically relevant ionic and stimulus paradigm conditions.

## METHODS

The mutations N999S (rs886039469), N999S/R1128W (rs747029218), and D434G (rs137853333) were introduced by site-directed mutagenesis into the WT human *KCNMA1* (BK channel) cDNA sequence (MG279689) in the pcDNA3.1+ mammalian expression vector. All mutations were verified by sequencing. Channel constructs contained an NH_2_-terminal extracellular Myc tag and enhanced yellow fluorescent protein (EYFP) in RCK2, used to identify transfected cells. Wild-type (WT) and mutant channel plasmids were transfected into HEK293T cells as previously described (Shelley et al. 2013), and electrophysiological recordings were performed 14–24 h after transfection.

BK currents were recorded in inside-out patch-clamp configuration at 22–24°C (Shelley et al. 2013). For symmetrical K^+^ recordings, electrodes (2–3 MΩ) were filled with (in mM) 140 KMeSO_3_, 2 KCl, 2 MgCl_2_, and 20 HEPES. The internal (bath) solution contained (in mM) 140 KMeSO_3_, 2 KCl, 20 HEPES, and 5 HEDTA with 1 μM CaCl_2_ (pH adjusted to 7.2 with KOH). In physiological K^+^ recordings, electrodes contained (in mM) 134 NaCl, 6 KCl, 1 MgCl_2_, 10 glucose, and 10 HEPES (pH 7.4 with NaOH). The internal (bath) solution contained (in mM) 110 KMeSO_3_, 10 NaCl, 30 KCl, 10 HEPES, 1 MgCl_2_, and 5 HEDTA with 10 µM CaCl_2_ (pH 7.2 with KOH). Free Ca^2+^ concentrations were calculated with WebMaxC (https://somapp.ucdmc.ucdavis.edu/pharmacology/bers/maxchelator/webmaxc/webmaxcS.htm).

Voltage commands were applied with a MultiClamp700B amplifier, Digidata1440, and Clampex v10.3 (Molecular Devices, Sunnyvale, CA). Macroscopic BK current traces were acquired at 50 kHz and filtered online at 10 kHz. Current parameters were analyzed in pCLAMP 10.6 (Molecular Devices). In each set of experimental conditions, two different voltage-step protocols were used. The first protocol was used to generate the conductance-voltage (*G-V*) relationship, the half-maximal voltage of activation (*V*_1/2_), and activation kinetics. The second protocol was applied to obtain deactivation kinetics. In symmetrical K^+^-1 μM Ca^2+^ experiments, the voltage activation protocol stepped from a holding potential (*V*_hold_) of −100 mV to +250 mV (Δ +10-mV increments) for 30 ms and back to −100 mV for 15 ms to generate tail currents. In physiological K^+^-10 μM Ca^2+^ experiments, the protocol stepped from a *V*_hold_ of −150 mV to +150 mV (Δ +10-mV increments) for 30 ms and back to −150 mV for 15 ms to generate tail currents. *G* was calculated from tail currents, 150–200 µs after the peak, normalized to the highest conductance calculated for each patch (*G*/*G*_max_), and plotted against membrane potential (*V*) to generate conductance-voltage (*G*/*G*_max_-*V*) curves. *V*_1/2_ values were calculated from a Boltzmann fit of the *G*/*G*_max_-*V* relationship (Prism v8.2.0; GraphPad Software, San Diego, CA). Activation time constants (τ_act_) were obtained from the same voltage activation protocol by fitting a single standard exponential to the rising phase of the outward K^+^ current. The protocol used to determine the deactivation time constant (τ_deact_) was the same in both ionic conditions: BK currents were activated by a +200-mV voltage step for 20 ms, from −150 mV (*V*_hold_), followed by 15-ms voltage steps from −200 mV to −50 mV (Δ +10-mV increments). Deactivation kinetics were measured by fitting tail currents with single-exponential functions. In all protocols, leak current was subtracted with a P/5 protocol with a subsweep *V*_hold_ of −120 mV (Shelley et al. 2013). A third voltage protocol was used in the physiological K^+^-10 μM Ca^2+^ condition to evoke AP-activated BK currents. This protocol consisted of a representative neuronal AP waveform as a voltage command (Shelley et al. 2013). BK current was measured at the peak of the AP command and normalized to the maximum steady-state current evoked by a standard voltage activation protocol (between +70 and +100 mV).

Acetazolamide (ACTZ) (catalog no. A6011; Sigma-Aldrich) experiments were performed in physiological K^+^-10 μM Ca^2+^ solutions. The voltage protocol consisted of a 30-ms activation step, from −150 mV (*V*_hold_) to the *V*_1/2_ for either WT or N999S channels, 60 times at 5-s intervals. After a 1-min baseline, 50 μM ACTZ (in DMSO) was added and, 3 min later, 100 nM paxilline (catalog no. 2006; Tocris, Bristol, UK). Steady-state BK current levels were plotted over time before and after drug application.

Statistical analysis was performed in Prism v8.2 (GraphPad) and consisted of one-way ANOVA with Tukey post hoc test (*V*_1/2_ values and normalized AP-evoked comparisons), repeated-measures ANOVA with Bonferroni post hoc (activation/deactivation kinetics), and paired *t* tests (before/after ACTZ). All data in figures are presented as means ± SE, with individual data points superimposed for *V*_1/2_ and AP values.

## RESULTS

### 

#### GOF properties of N999S channels.

Because of the lack of a BK channel splice variant with known physiological function cloned from human tissue, in this study the effects of WT and patient *KCNMA1* mutations were examined within the context of a brain variant cloned from mouse hypothalamus that was humanized (MG279689) (Shelley et al. 2013). The alternative exons contained within the gating ring are SRKR (RCK1/site 1), no exon (STREX/site 2), 27-aa exon (Ca^2+^ bowl/site 3), and the COOH-terminal exon RKEMVYR (Plante et al. 2019; Shelley et al. 2013), similar to human variant NM_001322835. This BK variant also contains an extracellular NH_2_-terminal Myc tag and EYFP in RCK2. Currents from this BK channel variant with and without these tags were similar (Plante et al. 2019).

To evaluate the effect of the N999S mutation on BK current properties, we recorded from inside-out patches from HEK293T cells transfected with WT or N999S channels. In neurons, the activation of BK is driven by the combination of voltage and an increase in intracellular Ca^2+^ level, and these experiments were therefore performed in the presence of 1 μM intracellular Ca^2+^ (Yang et al. 2015). With a voltage-clamp protocol of square depolarizing voltage steps, the currents from N999S channels were compared to WT channels across a range of voltages from −100 to 250 mV (Fig. 1*B*).

The normalized conductance-voltage (*G-V*) relationships were compared, which allows assessment of the relative voltage dependence of activation for mutant versus WT channels, as well as the kinetics of opening and closing. N999S channels were more sensitive to membrane depolarization (Fig. 1*C*). The voltage of half-maximal activation (*V*_1/2_) for N999S currents was shifted to more hyperpolarized potentials by 55 mV compared with WT (N999S: +78 ± 1.5 mV and WT: +133 ± 1.7 mV; Fig. 1*D*). However, no significant differences in the *G-V* slope were observed (WT *z* = 0.8 ± 0.01 vs. N999S *z* = 0.9 ± 0.07; *P* = 0.086, unpaired *t* test), suggesting that WT and N999S channel activations have similar voltage sensitivity.

Analysis of activation kinetics (τ_act_) between +120 and +170 mV showed a faster N999S activation than WT (Fig. 1*E*, *inset* at +160 mV). This difference is mediated by the shifted voltage dependence of N999S channels, which is 55 mV more hyperpolarized than WT, and can be demonstrated by analyzing voltages corrected for the *V*_1/2_ (τ_act_ at 120 mV, WT = 7.4 ± 1.5 and N999S = 8.04 ± 1.04; *P* = 0.25). Between +180 and +250 mV, where the channels are maximally open, the activation kinetic values between the currents were not different (Fig. 1*E*, *inset* at +240 mV). Contrary to what was observed with activation, N999S channels close slower (τ_deact_) across all voltages compared with WT. Between −60 and −100 mV, τ_deact_ values for N999S currents were ~5 times slower than those for WT (Fig. 1*F*). Taking these data together, the changes in BK current establish that the N999S mutation confers GOF properties to a brain-expressed BK channel variant, producing larger BK currents that activate at lower voltages because of the shifted *V*_1/2_ and activate faster and deactivate slower than WT.

**Fig. 2.**
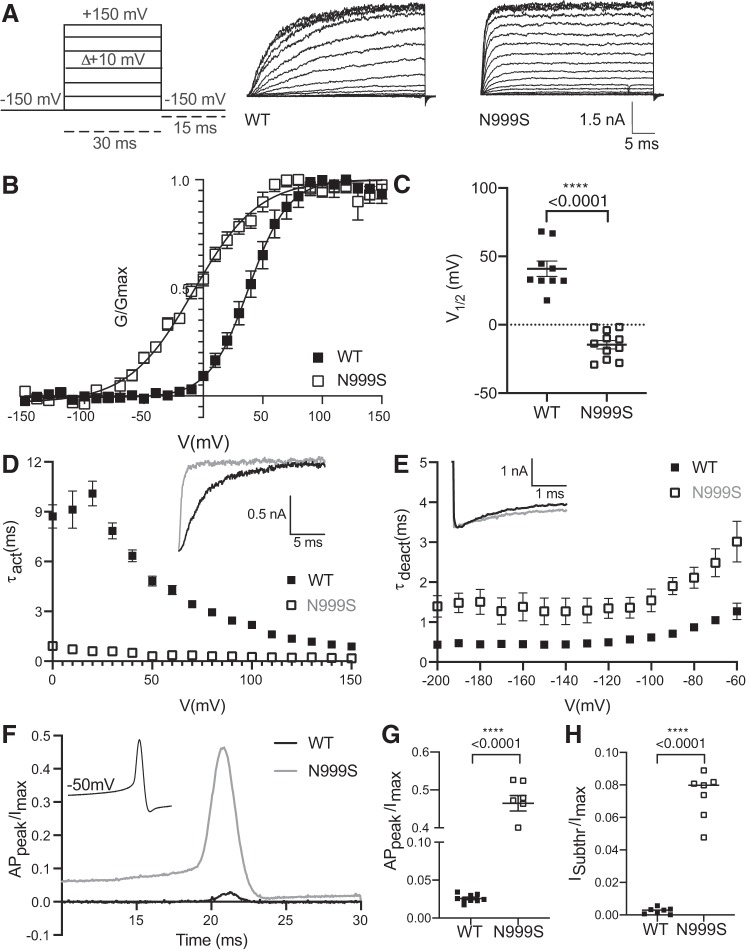
Wild-type (WT) and N999S channel properties in physiological K^+^ and 10 μM Ca^2+^. *A*: representative macroscopic currents at 10-mV increments are shown together with the voltage-step protocol applied. *B*: conductance-voltage (*G-V*) curves of WT (*n* = 9) and N999S (*n* = 11) channels. *G* was normalized to the highest conductance calculated (*G*_max_). *C*: half-maximal voltage of activation (*V*_1/2_) individual values for WT and N999S channels (*P* < 0.0001, 1-way ANOVA, Tukey post hoc test). *D*: activation time constant (τ_act_) vs. voltage for WT (*n* = 9) vs. N999S (*n* = 7). *Inset* shows representative activation traces of both channels at +100 mV. The N999S channels open significantly faster than the WT in a range between 0 and 100 mV (*P* < 0.0001, 2-way ANOVA with Bonferroni post hoc). *E*: comparison of WT (*n* = 8) and N999S (*n* = 6) deactivation time constants (τ_deact_). N999S exhibits slower deactivation kinetics compared with WT in the range of −80 to −60 mV (*P* < 0.05, 2-way ANOVAs with Bonferroni post hoc). *Inset* illustrates representative portion of the tail currents at −60 mV (2 s of the total 20-s deactivation protocol). *F*: action potential (AP) waveform voltage command (*inset*) and representative traces for WT and N999S AP-evoked currents. AP_peak_, AP peak current. *G*: normalized AP_peak_ level to the maximum steady-state current evoked by a standard voltage activation protocol (*I*_max_) (WT: 0.025, *n* = 9 and N999S: 0.465, *n* = 7; *P* < 0.0001, 2-way ANOVA, Tukey post hoc test). *H*: normalized subthreshold current *I*_Subthr_ level at −50 mV to *I*_max_ (WT: 0.002 ± 0.0007 and N999S: 0.07 ± 0.0008); ****P < 0.0001, 1-way ANOVA, Tukey post hoc test.

We next studied the N999S GOF phenotype in physiological Na^+^ and K^+^ gradients and 10 μM intracellular Ca^2+^ conditions, which mimics the K^+^-driving force on channel activation and intracellular Ca^2+^ levels during some types of neuronal activity (Fig. 2*A*). Because the BK channel is coupled to voltage-gated Ca^2+^ channels (Ca_v_) in neurons, 10 μM Ca^2+^ is the estimated average activating concentration during an AP (Fakler and Adelman 2008; Marrion and Tavalin 1998). N999S currents showed a 46-mV shift in the *V*_1/2_ toward negative potentials compared with WT in response to the protocol of depolarizing voltage steps (*V*_1/2_ values: N999S −8 ± 4.2 mV and WT +38 ± 1.4 mV; Fig. 2, *B* and *C*). Thus N999S channels are significantly more active at more hyperpolarized potentials compared with WT, resulting in larger outward K^+^ currents (Fig. 2*B*). Interestingly, under these conditions N999S shows a paradoxical reduction in voltage sensitivity (slope, *z* = 0.95 ± 0.05) compared with WT (*z* = 1.56 ± 0.17; *P* = 0.0002, unpaired *t* test), despite its GOF effect on channel activity.

The effects of the mutation on activation of the channels was more pronounced under physiological K^+^-10 μM Ca^2+^. N999S channels opened 5 times faster (τ_act_) between 0 and +100 mV compared with WT (Fig. 2*D*), whereas N999S channels transitioned to the closed state (τ_deact_) ~4 times slower than the WT channels between −80 and −60 mV (Fig. 2*E*). These data corroborate a strong GOF phenotype under physiological K^+^ conditions, with changes in channel properties similar to those observed previously in symmetrical K^+^ and lower (1 μM) Ca^2+^.

The relevance of the N999S GOF phenotype in physiological K^+^-10 μM Ca^2+^ conditions was further probed by applying a dynamic voltage command obtained from a central neuron (Plante et al. 2019; Wang et al. 2009). Using this AP waveform as a voltage stimulus, we observed a massive increase of 18-fold in the AP-evoked current amplitude from N999S channels compared with WT (Fig. 2, *F* and *G*). Additionally, preceding the upstroke of the AP, the N999S subthreshold current is 35 times larger than WT measured at −50 mV (Fig. 2*H*). Considering these data, it is likely that the GOF properties conferred by N999S would affect BK currents in a typical neuronal context, both increasing the subthreshold K^+^ current that could oppose initiation of the AP as well as increasing the amount of K^+^ current evoked during an AP.

#### N999S GOF activity compared to D434G channels.

**Fig. 3.**
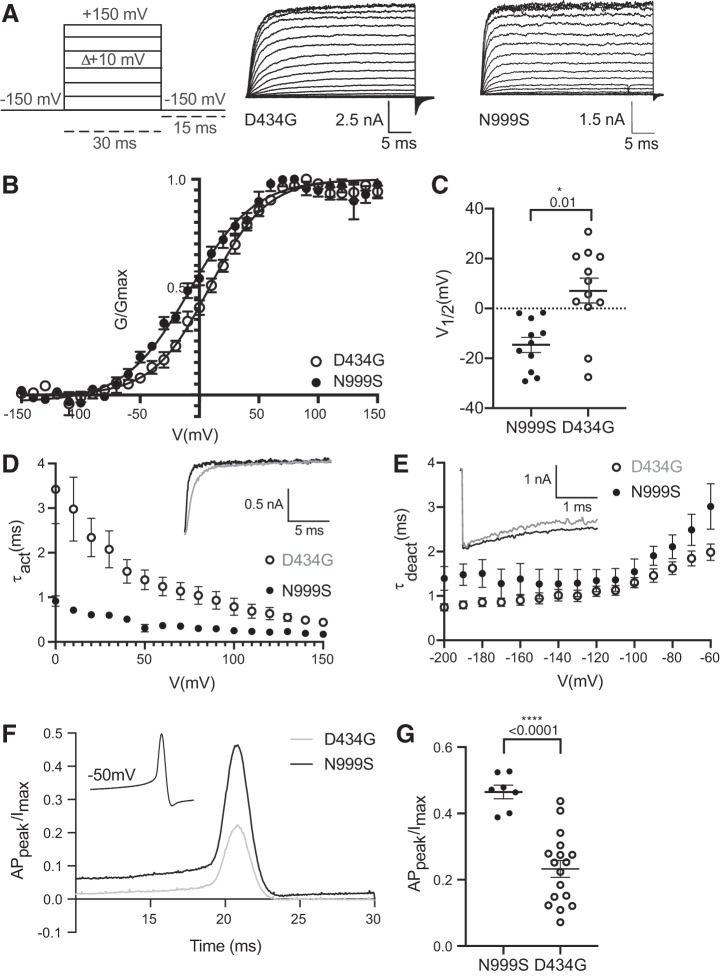
Comparison between N999S and D434G channel properties in physiological K^+^ and 10 μM Ca^2+^. *A*: representative macroscopic currents with the voltage-step protocol. *B*: conductance-voltage (*G-V*) relationships of N999S (*n* = 11) and D434G (*n* = 12) channels. The *G-V* curves are fitted by a Boltzmann function (solid line) with a half-maximal voltage of activation (*V*_1/2_) of −8 ± 4.2 mV for N999S and +8 ± 3.2 mV for D434G mutations. *G* was normalized to the highest conductance calculated (*G*_max_). *C*: individual *V*_1/2_ values for N999S and D434G are significantly different (*P = 0.01, 1-way ANOVA with Tukey post hoc). *D*: activation kinetics [activation time constant (τ_act_)] of N999S (*n* = 7) and D434G (*n* = 12) mutations. *Inset* corresponds to representative activation traces of both channels at +100 mV. The N999S channels open faster than D434G in the voltage range between 0 and 100 mV (*P* < 0.0001, 2-way ANOVA with Bonferroni post hoc). *E*: deactivation kinetics [deactivation time constant (τ_deact_)] of N999S (*n* = 6) vs. D434G (*n* = 10). The N999S mutation has slower deactivation kinetics than the D434G mutation only at −60 mV (*P* < 0.0001, 2-way ANOVA with Bonferroni post hoc). *Inset* illustrates a representative portion of the tail currents at −60 mV (2 s of the total 20-s deactivation protocol). *F*: voltage protocol and representative traces of N999S and D434G action potential (AP)-evoked current. AP_peak_, AP peak current. *G*: normalized AP_peak_ values to the maximum steady-state current evoked by a standard voltage activation protocol (*I*_max_) for both mutations. The amplitude of N999S (*n* = 7) current is 2 times larger than that of D434G (*n* = 12) (****P < 0.0001, 1-way ANOVA with Tukey post hoc test).

To evaluate the degree of GOF activity for N999S, we compared it to the first identified and more extensively studied *KCNMA1* GOF mutation, D434G (Du et al. 2005; Wang et al. 2009; Yang et al. 2010). Previous studies of the D434G mutation show a *V*_1/2_ shifted to negative potentials, faster activation, slower deactivation, an increase in open probability, and an increased Ca^2+^ sensitivity compared with the WT BK currents (Díez-Sampedro et al. 2006; Du et al. 2005; Plante et al. 2019; Wang et al. 2009; Yang et al. 2010). Consistent with previous studies, in physiological K^+^ and 10 μM Ca^2+^ conditions (Wang et al. 2009), D434G had GOF current properties with a *V*_1/2_ of +8 ± 3.2 mV (Fig. 3, *A–C*). However, N999S currents were still 17 mV more shifted to negative potentials (*V*_1/2_ −8 ± 4.2 mV). Although activation of D434G was already faster than WT (at +30 mV WT τ_act_ = 7.8 ms vs. D434G τ_act_ = 2.1 ms; Figs. 2*D* and 3*D*), activation of N999S was 3.5 times faster than D434G between 0 and 100 mV. Similarly, even though D434G channels already closed slower than WT (at −60 mV WT τ_deact_ = 1.2 ms vs. D434G τ_deact_ = 1.76 ms; Figs. 2*E* and 3*E*), the τ_deact_ for N999S was still 4 times slower than D434G at −60 mV. Because N999S exceeds the GOF phenotype of D434G with respect to the voltage dependence of activation, and channels activate faster and deactivate slower, this suggests a greater potential for N999S to alter neuronal excitability.

To test this, the range of voltages over which the differences between N999S and D434G were expressed was considered. The neuronal resting membrane potential is between −70 and −50 mV, whereas the peak of the AP happens between 10 and 50 mV, depending on the neuronal type (Bean 2007). Because the differences in biophysical properties between N999S and D434G manifest in this range of voltages, we evaluated the AP-evoked BK currents generated by these two mutations. Although the AP-evoked current from D434G channels was already 9 times greater than WT (Fig. 2, *F* and *G*, and Fig. 3, *F* and *G*), the AP-evoked current generated by N999S channels was approximately twice as large as that of D434G. Thus the biophysical GOF alterations caused by the N999S mutation surpass D434G in the ability to generate larger AP-evoked currents, suggesting the potential for a distinct, and presumably stronger, GOF phenotype in vivo.

#### Effect of second-site mutation R1128W.

**Fig. 4.**
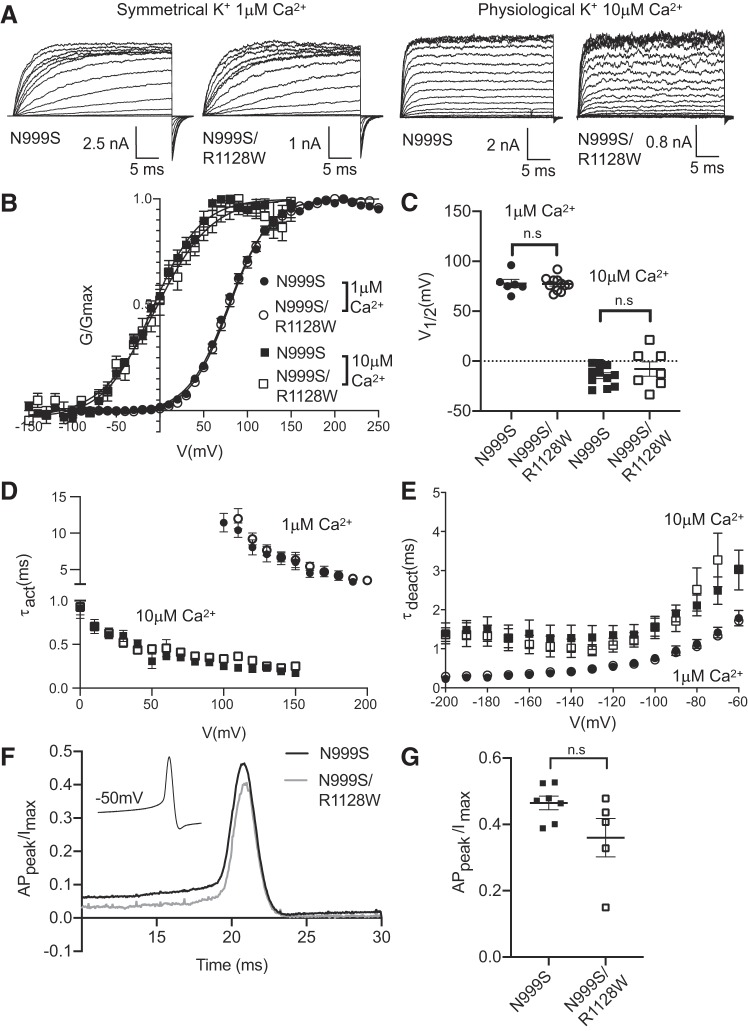
Effect of the R1128W mutation with N999S. *A*: representative macroscopic currents at +20-mV increments for 1 μM Ca^2+^ or +10 mV with 10 μM Ca^2+^ are depicted. *B*: conductance-voltage (*G-V*) relationships of N999S (*n* = 11) and N999S/R1128W (*n* = 7) channels in symmetrical K^+^ and 1 μM Ca^2+^ and physiological K^+^ and 10 μM Ca^2+^. *G* was normalized to the highest conductance calculated (*G*_max_). The N999S/R1128W half-maximal voltage of activation (*V*_1/2_) is +79 ± 1 mV in symmetrical K^+^-1 μM Ca^2+^ and −4 ± 6 mV in physiological K^+^-10 μM Ca^2+^. *C*: individual *V*_1/2_ values for N999S and N999S/R1128W are not statistically different [*P* > 0.05 (n.s.), 1-way ANOVA with Tukey post hoc test]. *D* and *E*: activation [*D*; activation time constant (τ_act_) N999S/R1128W *n* = 5 and N999S *n* = 7] and deactivation [*E*, deactivation time constant (τ_deact_) N999S/R1128W *n* = 5 and N999S *n* = 7] kinetics of N999S and N999S/R1128W mutations. There is no significant difference in both kinetics in the entire range of voltages evaluated (*P* > 0.05, 2-way ANOVA with Bonferroni post hoc). *F*: voltage protocol (*inset*) and representative traces of N999S and N999S/R1128W action potential (AP)-evoked current. AP_peak_, AP peak current. *G*: normalized AP_peak_ values for both mutations (N999S/R1128W *n* = 5 and N999S *n* = 7) show the same current amplitude, [*P* > 0.05 (n.s.), 1-way ANOVA with Tukey post hoc test].

One of the three patients described recently harbors a second de novo mutation, R1128W, in addition to N999S (Heim et al. 2019). To determine whether the N999S/R1128W double mutation presents a phenotype different from N999S alone, we evaluated the biophysical properties of N999S/R1128W double-mutant channels in symmetrical K^+^-1 μM Ca^2+^ and physiological K^+^-10 μM Ca^2+^ conditions (Fig. 4*A*). We observed no significant differences in the *V*_1/2_ between N999S and N999S/R1128W in both conditions (Fig. 4, *B* and *C*). Both types of channels exhibit the same activation and deactivation kinetics, with no significant differences between them (Fig. 4, *D* and *E*). Finally, when we evaluated the impact of the N999S/R1128W double mutation on the AP-evoked currents we observed no significant difference compared with the N999S mutation alone (Fig. 4, *F* and *G*). Thus we conclude that there is no additional phenotype in the N999S/R1128W double mutation, neither exacerbating nor mitigating the primary alteration produced by N999S. This finding agrees with the lack of additional symptoms in this patient compared with patients harboring N999S alone (Heim et al. 2019). However, it is still possible that the phenotype of N999S mutation masks a phenotype associated with the R1128W mutation alone that is not expressed under the conditions tested here.

#### Effect of acetazolamide in N999S and WT BK channels.

**Fig. 5.**
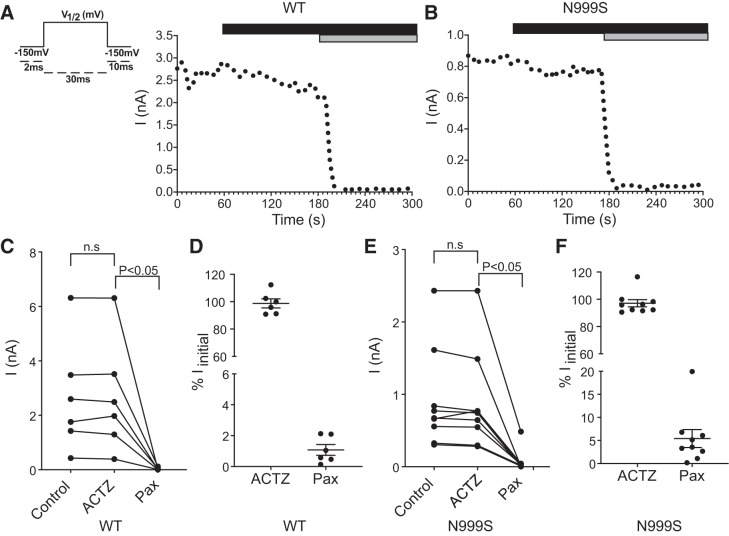
Effect of acetazolamide (ACTZ) and paxilline on wild-type (WT) and N999S channels. *A* and *B*: representative experimental protocol (*left*) and current (*I*) level vs. time for WT (*A*) and N999S (*B*) channels. The black bar denotes application of 50 μM ACTZ to the patches, and the gray bar corresponds to application of 100 nM paxilline. *C* and *E*: individual current levels from each patch before ACTZ (30 s), after ACTZ (120 s), and after paxilline (Pax; 240 s) for WT (*n* = 6) and N999S (*n* = 9) channels. *D* and *F*: normalized data presented as % of the initial current (*I*_initial_). No significant differences were found for WT or N999S channels after 50 μM ACTZ [*P* > 0.05 (n.s.), paired *t* test]. The reduction of the current due to paxilline application was significant (*P* < 0.05, paired *t* test).

Acetazolamide (ACTZ), an inhibitor of the enzyme carbonic anhydrase, is a drug widely used in epilepsy treatment (Reiss and Oles 1996; Wada et al. 1961). One of the three patients harboring the N999S mutation referred to in this study responded positively to the ACTZ treatment (Heim et al. 2019). However, in addition to the carbonic anhydrase mechanism, ACTZ has been reported to have a direct agonist effect on BK channels in skeletal muscle (Dinardo et al. 2012; Tricarico et al. 2004). Given the possibility of exacerbation of GOF activity by exposure to an agonist, which could worsen patient symptoms, we studied the effect of ACTZ on N999S and WT channels in the brain splice variant background. The protocol used consisted of a 20-ms depolarizing pulse at the N999S or WT channel’s *V*_1/2_ every 5 s. After a 1-min baseline, 50 μM ACTZ was applied and the steady-state current sizes were evaluated (Fig. 5, *A* and *B*). The concentration of 50 μM was calculated as an estimation of the plasma concentration for an antiepileptic-dose treatment of 250 mg/day, with a clearance of 36.7 mL/min (Yano et al. 1998). Additionally, the concentration reported to activate BK channels in vitro ranged from 10 to 200 μM (Dinardo et al. 2012; Tricarico et al. 2004). First, we found no significant effect of ACTZ on the current magnitude for WT channels (Fig. 5, *A*, *C*, and *D*), establishing that ACTZ does not show agonist activity under these conditions. N999S channels also did not respond to ACTZ application, showing neither an increase in activity nor a difference compared with WT (Fig. 5, *B*, *E*, and *F*). As a perfusion control, the BK channel inhibitor paxilline (100 nM) was applied at the end of the experiments. Paxilline was able to inhibit 98% of WT and 95% of N999S BK currents from the patches (*P* > 0.05, 1-way ANOVA). Reduction in paxilline’s effect on N999S channels has been reported, but the effect was less in this study than previously observed (Li et al. 2018). Taken together, this experiment demonstrates that ACTZ has no direct agonist effect on the WT splice variant used in this study and, furthermore, N999S channels do not differ from WT in their lack of response to ACTZ.

## DISCUSSION

In this study, the properties of BK channels encoded by *KCNMA1* patient mutations associated with epilepsy and/or paroxysmal nonkinesigenic dyskinesia (PNKD) were investigated. Electrophysiological characterization of N999S, N999S/R1128W, and D434G BK currents demonstrated that each mutation manifests strong GOF properties on a brain splice variant background with AP stimuli compared with WT channels. Comparison of the currents revealed N999S to have greater effects on channel properties compared with the previously characterized D434G mutation, further shifting the voltage dependence of activation to hyperpolarized potentials and further speeding activation and slowing deactivation of the channels. We additionally showed that neither the second-site mutation R1128W nor the antiepileptic ACTZ exacerbated the primary N999S GOF current phenotype.

Interpretation of the effect of the mutations on BK current properties is qualified by the presence of a Myc tag at the NH_2_ terminus and a YFP tag inserted at the beginning of the RCK2 domain. However, the *V*_1/2_s produced by tagged and untagged WT channels with the same splice variant is similar at 0, 1, 10, and 100 μM Ca^2+^ (Plante et al. 2019), suggesting that the tags do not alter BK channel function. Additionally, the results here agree with other studies reporting D434G and N999S current properties using untagged channel variants, including the direction and order of magnitude of the *V*_1/2_ shift and the ∼3 times faster activation for D434G (activation was not studied previously for N999S) (Li et al. 2018; Wang et al. 2009; Yang et al. 2010). Some minor variations in the magnitude of the *V*_1/2_ shifts between this and the other studies could be due to the different Ca^2+^ concentrations and splice variants used. Additionally, green fluorescent protein insertions at several positions in the linker between RCK1 and RCK2 do not show significant differences in the *V*_1/2_ values, either (Giraldez et al. 2005), suggesting that this region of the channel tolerates insertion of fluorescent proteins without substantial perturbations in the gating properties. Although small effects from the tags can become relevant in detailed biophysical characterization of these mutations, the consistency with other studies using WT and mutant untagged channels supports the comparative interpretation of the N999S and D434G current properties presented here.

The mechanism by which N999S alters BK channel activity is not fully elucidated by these data, but several lines of evidence suggest that this mutation primarily alters voltage-dependent gating. The similar magnitude of the *V*_1/2_ shift for N999S compared with WT in Ca^2+^-free conditions (data not shown), and at both 1 and 10 μM Ca^2+^, suggests no change over the Ca^2+^ gating mechanism. This observation is consistent with prior studies showing that mutations of the Ca^2+^-binding sites in the gating ring do not eliminate the increase in current conferred by N999S (Li et al. 2018). Interestingly, at 10 μM Ca^2+^ a change in the slope of the *G-V* relationship was observed, suggesting a small effect on the voltage activation mechanism. Considering the distal location of N999S at the end of the COOH terminus (Fig. 1*A*), this change in slope could reflect an alteration in the allosteric gating mechanism through the VSD domain. The substitution of serine for asparagine, which retains the polar properties, could confer a new potential for phosphorylation at that residue. However, Netphos analysis does not show any consensus kinase site in this region with the mutation, suggesting that other properties distinguishing these residues factor into the differences. Interestingly, despite its distal localization in the primary amino acid sequence, the N999 residue is near the AC region that connects TM6 with the gating ring in the open-state BK channel crystal structure (*Aplysia* Slo1). This AC region is essential for coupling in the allosteric gating mechanism (Tao et al. 2017). The D434G mutation is also located in the AC region, but near the Ca^2+^-binding site of RCK1 (Fig. 1*A*), which, in contrast to N999S, is consistent with alteration of the Ca^2+^ gating mechanism by D434G (Li et al. 2018; Wang et al. 2009; Yang et al. 2010). Even more distally located at the end of the COOH terminus, the R1128W mutation would result in a nonconservative loss of a positively charged arginine, replaced by the bulky hydrophobic tryptophan. However, R1128 is not resolved in the two crystal structures currently available (Tao et al. 2017; Yuan et al. 2010), and little is known about the role of residues near R1128 in BK channel gating. Thus, despite the GOF nature of both the D434G and N999S mutations, the potentially distinct biophysical mechanisms produce a different magnitude of effect on BK current properties that is detectable under one type of reconstituted physiological condition. In this study, the N999S mutation clearly surpasses the D434G GOF phenotype, with a more negative *V*_1/2_ and faster activation and slower deactivation kinetics. These differences resulted in larger AP-evoked currents from N999S channels compared with D434G, yet patients harboring N999S, N999S/R1128W, and D434G GOF mutations share largely the same core symptoms of epilepsy and/or PNKD, suggesting a mechanistically similar effect on excitability in vivo.

On one hand, these core symptoms may actually be more variable when examined in detail. Closer scrutiny of patient phenotypes reveals there is variability in the prevalence and type of seizure and the prevalence of dyskinesia, both between D434G and N999S patients and also within each mutation group (Bailey et al. 2019). The variability could stem from differences in the genetic backgrounds between patients, consistent with the de novo nature of the N999S mutations, but it is also observed in related individuals harboring the D434G mutation. Interestingly, some of the patients harboring the N999S mutations also present developmental delay and/or intellectual disability, a condition not reported for D434G patients (Bailey et al. 2019). The possibility that distinct symptoms could be associated with each GOF mutation can be explored further as more patients are identified and reported with standardized diagnostic criteria.

On the other hand, it is possible that in the neuronal context these two GOF mutations produce more similar effects on BK currents than studies in heterologous cells reveal. The differences between the mutations in vivo could be reduced by coassembly with WT and accessory subunits, or at physiological temperatures and patterns of AP firing that were not tested in this study. These experiments were performed on homotetrameric channels because this represents the most controlled condition with which to directly compare the WT and mutant BK current types. However, patients harboring D434G and N999S are heterozygous (Du et al. 2005; Heim et al. 2019; Li et al. 2018), which leads to the possibility of BK channels composed of WT/N999S and WT/D434G heterotetramers that could potentially attenuate the GOF phenotype. At present, no information is available about the allele expression that could give clues about the stoichiometry of patient channels in native cells. Besides heterotetramerization, other possibilities that could reduce the difference between these two mutations in vivo include that the N999S and D434G mutations could produce different magnitude effects according to the splice variants expressed. Across tissues or between different neuronal subtypes, the distinct BK channel splice variants show differences in channel activity, Ca^2+^ sensitivity, activation and deactivation kinetics, and interaction with accessory subunits (Chen et al. 2005; Latorre et al. 2017). However, the substantial shift in the voltage dependence of activation for N999S channels found in this study is similar to the 59-mV shift reported with a different BK channel splice variant (Li et al. 2018), suggesting that the GOF effects are effectively conferred through at least some sequence variations in the BKα subunit. Other factors in the native neuronal context could also mitigate the phenotype and render N999S channels more similar to D434G or even closer to WT in their properties. One contributor to this possibility could be coexpression with an accessory subunit, such as the neuronal β4 subunit. β4 slows BK channel activation and deactivation and shifts the voltage dependence of activation, with a net effect of reducing AP-evoked BK current (Brenner et al. 2000; Wang et al. 2006). Nevertheless, coexpression of N999S or D434G channels with β4 produces a effect on those currents similar to the effect on WT, with the mutant currents remaining left-shifted and faster activating compared with WT currents (Li et al. 2018; Wang et al. 2009). For D434G + β4, this results in larger AP-evoked currents compared with WT (Wang et al. 2009). These studies suggest that inclusion of β4 in mutant channel complexes might not prevent either the increased current compared with WT or the increased current from N999S compared with D434G in vivo. Finally, firing patterns and ionic conditions different from those tested here could also serve to reduce differences between N999S and D434G. For example, under a train of APs a sustained elevation of intracellular Ca^2+^ could maximally activate both types of mutant channels and collapse the differences in evoked current between them. This was partly observed for D434G currents, where the difference compared with WT was reduced above 30 μM Ca^2+^ until it overlapped at 100 μM Ca^2+^ (Wang et al. 2009; Yang et al. 2010).

Because the N999S and N999S/R1128W mutations are associated with a high frequency of seizures and PNKD attacks in patients, it is important to understand under what conditions the mutations might express their most dramatic effects on excitability. Our data suggest two possible changes in membrane excitability associated with the mutations that could lead to alterations in neuronal activity. At the neuronal resting potential, between −70 and −50 mV, depending on the neuronal type (Bean 2007), the voltage-activated Ca^2+^ channels have a significant open probability. This allows the local Ca^2+^ concentration to increase dynamically in a range between 1 and 50 μM Ca^2+^, which activates the BK channels (Contreras et al. 2013; Fakler and Adelman 2008; Marrion and Tavalin 1998; Müller et al. 2007). In neurons expressing BK channels with the N999S mutation, the first potential mechanism to change neuronal excitability would be via a decrease in firing due to increased subthreshold BK current. This increased subthreshold current would result from the significantly shifted voltage dependence of activation for N999S channels to more hyperpolarized potentials. Our results show that N999S channels are 35 times more active than WT at subthreshold potentials at 10 μΜ calcium. In this case, firing would decrease because it would be harder to exceed threshold, making triggering the AP more difficult. If this occurred in inhibitory neurons, the reduced activity could precipitate seizure. A second potential change in excitability could be produced by increased N999S current during the AP, when the threshold is surpassed. Under voltage clamp, AP-evoked currents from N999S channels were 18-fold larger at the peak compared with WT. This larger current could contribute to changing firing in either direction, depending on the amount of current activated during repolarization versus the afterhyperpolarization (AHP) (Contet et al. 2016; Montgomery and Meredith 2012). For example, in the simplest cases, firing would be expected to increase if N999S contributed predominantly to repolarizing current, whereas decreased firing could be obtained if the AHP were prolonged. Because of both the faster activation and the slower deactivation, it is likely that N999S channels would contribute to both phases, making the net effect on AP activity uncertain until defined within a specific neuronal context and firing pattern.

Finally, when seizure activity is provoked, the efficacy of drug treatment could be affected by the potential for off-target effects on ion channels such as BK. The widely used antiepileptic drug acetazolamide (ACTZ) (Reiss and Oles 1996; Wada et al. 1961) was administered to one of the three patients harboring the N999S mutations, showing a positive response (Heim et al. 2019). ACTZ has a direct effect on carbonic anhydrase and several off-target effects, but most notably it has also been previously reported as a direct agonist on skeletal muscle splice variant BK channels (Dinardo et al. 2012; Tricarico et al. 2004), raising the possibility that it would exacerbate GOF BK channel activity. However, our data show that ACTZ has no effect on either WT or N999S channels on the brain variant, suggesting that it would not exacerbate the pathogenicity of the GOF properties in vivo. Nevertheless, it is possible that this lack of agonist effect could be due to the different splice variant expressed in skeletal muscle cells, which differs from the variant in this study at alternate splice site 2 (STREX) and the sequence of exon 21 and intron 22 (Dinardo et al. 2012), or the lack of other channel components in the heterologous versus native cell context. However, since the N999S mutation already produces an increase in BK channel activity, any agonist effect of ACTZ is unlikely to be the mechanism causing the positive response in the patient.

## GRANTS

This work was supported by grants from NHLBI (R01 HL-102758, A. L. Meredith) and the Training Program in Integrative Membrane Biology, NIGMS (T32 GM-008181, A. L. Meredith) and The American Physiological Society’s Ryuji Ueno award, sponsored by the S & R Foundation (A. L. Meredith).

## DISCLOSURES

No conflicts of interest, financial or otherwise, are declared by the authors.

## AUTHOR CONTRIBUTIONS

H.J.M. and A.L.M. conceived and designed research; H.J.M. and K.K.M. performed experiments; H.J.M., K.K.M., and A.L.M. analyzed data; H.J.M., K.K.M., and A.L.M. interpreted results of experiments; H.J.M. prepared figures; H.J.M. and A.L.M. drafted manuscript; H.J.M., K.K.M., and A.L.M. edited and revised manuscript; H.J.M., K.K.M., and A.L.M. approved final version of manuscript.
